# The Persistence of Bacterial Pathogens in Surface Water and Its Impact on Global Food Safety

**DOI:** 10.3390/pathogens10111391

**Published:** 2021-10-27

**Authors:** Rebecca L. Bell, Julie A. Kase, Lisa M. Harrison, Kannan V. Balan, Uma Babu, Yi Chen, Dumitru Macarisin, Hee Jin Kwon, Jie Zheng, Eric L. Stevens, Jianghong Meng, Eric W. Brown

**Affiliations:** 1 Office of Regulatory Science, Center for Food Safety and Applied Nutrition, Food and Drug Administration, College Park, MD 20740, USA; julie.kase@fda.hhs.gov (J.A.K.); yi.chen@fda.hhs.gov (Y.C.); dumitru.macarisin@fda.hhs.gov (D.M.); heejin.kwon@fda.hhs.gov (H.J.K.); jie.zheng@fda.hhs.gov (J.Z.); eric.brown@fda.hhs.gov (E.W.B.); 2 Office of Applied Research and Safety Assessment, Center for Food Safety and Applied Nutrition, Food and Drug Administration, Laurel, MD 20708, USA; lisa.plemons@fda.hhs.gov (L.M.H.); kannan.balan@fda.hhs.gov (K.V.B.); uma.babu@fda.hhs.gov (U.B.); 3 Office of the Center Director, Center for Food Safety and Applied Nutrition, Food and Drug Administration, College Park, MD 20740, USA; eric.stevens@fda.hhs.gov; 4 Joint Institute for Food Safety and Applied Nutrition, Center for Food Safety and Security Systems, University of Maryland, College Park, MD 20742, USA; jmeng@umd.edu

**Keywords:** Shiga toxin-producing *E. coli*, *Salmonella*, *Campylobacter*, *Listeria monocytogenes*, water, antimicrobial resistance, global initiatives

## Abstract

Water is vital to agriculture. It is essential that the water used for the production of fresh produce commodities be safe. Microbial pathogens are able to survive for extended periods of time in water. It is critical to understand their biology and ecology in this ecosystem in order to develop better mitigation strategies for farmers who grow these food crops. In this review the prevalence, persistence and ecology of four major foodborne pathogens, Shiga toxin-producing *Escherichia coli* (STEC), *Salmonella*, *Campylobacter* and closely related *Arcobacter*, and *Listeria monocytogenes*, in water are discussed. These pathogens have been linked to fresh produce outbreaks, some with devastating consequences, where, in a few cases, the contamination event has been traced to water used for crop production or post-harvest activities. In addition, antimicrobial resistance, methods improvements, including the role of genomics in aiding in the understanding of these pathogens, are discussed. Finally, global initiatives to improve our knowledge base of these pathogens around the world are touched upon.

## 1. Introduction

Foodborne bacterial pathogens are microbes that when they contaminate food can lead to illness, typically gastroenteritis. The top four foodborne bacterial pathogens in the U.S. and Europe are Shiga toxin-producing *E. coli* (STEC), *Salmonella*, *Campylobacter* and *Listeria monocytogenes* [1,2]. Any food can become contaminated but of particular concern are fresh produce commodities that do not undergo a kill step before consumption. There are many points along the farm to fork continuum that may lead to the contamination of fresh produce but one of the most important routes is exposure from contaminated agricultural water. Agricultural water is defined as water that is intended to, or likely to, come into contact with food crops as it is used for all growing activities, including irrigation and washing/cooling of produce, as well as water used for preparing crop sprays, farm equipment cleaning, and dust abatement [3]. The EPA has defined standards for drinkable (i.e., potable) water and water for recreational use. However, to date, we do not have an implemented standard for safe agricultural water. The Produce Safety Rule had outlined a set of standards, but these have since been placed back under review [3]. These standards like the EPA standards are based on the counts of generic *E. coli* found in a set volume of agricultural water. However, as discussed in this review, pathogens are often present even when waters do not have an appreciable amount of generic *E. coli*.

Surface and agricultural waters are often surveyed for specific pathogens as part of longitudinal environmental studies focused on produce safety. Such studies have become commonplace in recent times. In particular, several longitudinal studies have centered on the long-term microbiological safety of tomatoes from *Salmonella* Newport and other highly adapted salmonellae on farm and surrounding environmental niches [4,5]. Additional substantial longitudinal efforts have reported on temporal and geographically disparate enteric pathogen persistence in surface and other agriculturally relevant waters (i.e., irrigation sources) around the U.S. [6,7,8]. Recent efforts focused on the importance of agricultural waters as potential reservoirs and vectors for enteric pathogen spread include (i) the CONSERVE waterborne pathogen consortium [9,10] and (ii) the numerous examples of surface and agricultural water related research from FDA’s Foods Program microbiologists and collaborators, focused heavily at the intersect between surface and agricultural waters and the environmental persistence of foodborne enteric pathogens including but not limited to *Salmonella*, enterohemorrhagic *E. coli*, and *L. monocytogenes* [11]. Parallel studies on enteric pathogen prevalence in water have also been reported in agriculturally rich areas of South America and other farm regions around the world [12].

In this review, the four major foodborne pathogens in relation to their presence, persistence and ecology in water are discussed. Also, the topics on antimicrobial resistance, method improvements and genomics are touched upon. Finally, an expanding international effort to understand these pathogens on a more global scale is highlighted.

## 2. The Pathogens

### 2.1. Pathogenic E. coli: O157 and Other Shiga Toxin-Producing E. coli (STEC)

Shiga toxin-producing *Escherichia coli* (STEC) are both food and water borne pathogens. Globally, it is estimated that half of the STEC disease risk is foodborne in origin [13]. The first symptoms are diarrhea and abdominal pain, but for some, the illness can progress into hemolytic uremic syndrome (HUS) and possibly death [14]. Among other factors, the severity or progression of symptoms may depend on the serotype of pathogenic STEC causing the infection. *E. coli* O157:H7 is the serotype most often associated with outbreaks of human illness but serogroups O26, O45, O103, O111, O121, and O145 are recognized as the top illness-causing non-O157 STECs in the U.S. [15,16] with the USDA FSIS ruling them as adulterants in both domestic and imported raw non-intact beef and beef products intended for non-intact use [17]. These six serogroups, along with O157:H7, form the U.S. “Top 7” enterohemorrhagic *E. coli* (EHEC) with a higher pathogenicity profile when strains possess intimin, an attaching and effacing protein encoded by *eae* (reviewed in [18]). EHECs also carry one or both Shiga toxin genes (*stx1*, *stx2*) and are a subset of the larger STEC group. Notably, the top illness-causing STEC serogroups shift depending upon geographical location. For example, Europe reports O157, O26, O103, O91 and O145 as the “Top 5” serogroups in human STEC infections [19].

In the U.S. and globally, STEC outbreaks have been linked to the consumption of leafy greens, sprouts, raw milk and cheeses, and raw beef and poultry [20,21,22]. Human illnesses involving raw beef and milk commodities are related to the fact that while many different animals can be hosts of STEC, ruminants, primarily cattle, are considered a major reservoir and contamination of cattle-sourced food products can occur [23]. STEC outbreaks involving leafy greens in the U.S. have been a recurring issue for *E. coli* O157:H7 since 1995 and non-O157 STEC since 2010 (reviewed in [24]) [22,25,26] with recurring O157:H7 outbreaks involving romaine lettuce occurring for the last several years [27,28]. The large, deadly 2006 multistate outbreak involving O157:H7 in bagged spinach launched federal and industry-driven initiatives targeting the improvement of leafy green vegetable safety which tightened standards for the microbial quality of irrigation water among other things [29]. Under the newly adopted Leafy Greens Marketing Agreement for water metrics in both California and Arizona, water applied by overhead irrigation 21 days or less before harvest and obtained from an open source, like a canal, must be treated to ensure no pathogens, such as STEC, are present. Testing is also required throughout the irrigation system to ensure the water continues to be pathogen-free (lgma.ca.gov; lgma.az.gov). Evidence from previous FDA outbreak investigations involving romaine lettuce suggests that agricultural waters may be a contributing factor in contaminating the lettuce or co-contaminated by the same STEC source, since water from irrigation canals, on-farm surface reservoirs, or other nearby surface water yielded either the particular outbreak strain or other *E. coli* O157:H7 strains [27,28,30]. 

From seed to consumption, sprouts are in constant contact with varying amounts of water. Germination is induced by soaking a seed, grain, nut, or bean. While the microbial quality of the water used is important, it is possible that the seeds themselves are contaminated and the subsequent warm, moist environment with ample organic matter for food, creates an ideal environment for *E. coli* proliferation with consumption very often occurring without a kill-step such as cooking [31]. Not surprisingly, sprouts have been repeatedly linked to outbreaks of STEC. Some outbreaks have occurred with the same strain annually, O103:H2 in clover sprouts (years 2019 and 2020) [32], others on a global scale, O104:H4 in fenugreek sprouts [33], with ill consumers in 16 countries. Notably, the 2011 *E. coli* O104:H4 outbreak remains one of the largest to date with an unusually high rate of HUS. In the U.S., sprout firms are covered by the FDA Produce Safety Rule which means that water used in a sprout operation must meet the no detectable generic *E. coli* per 100 ml microbial quality criterion [34]. Moreover, sprout-specific requirements exist that dictate the testing of spent sprout irrigation water for *E. coli* O157:H7. Similarly, in the E.U., when testing spent sprout water, it must be free of O157, O26, O111, O103, O145 and O104:H4 [35]. 

A limited number of STEC prevalence surveys have been conducted within U.S. surface and agricultural waters. Haymaker et al. (2019) found STEC in 2.35% of surface and reclaimed water samples collected from 10 sites in mid-Atlantic U.S. states [10]. Over a 12-month period, temporally and geographically, two sampling events yielded most of the STEC isolates. Similar findings were obtained from sampling the U.S. Great Lakes. Although *E. coli* isolates from Lake Erie were not confirmed as STEC, virulence gene analysis revealed that < 1% were EHEC [36]. A subsequent study focused on Lake Superior found no STEC or EHEC in 3557 *E. coli* strains examined [37]. Rivers and streams sampled from the Upper Oconee Watershed, a mixed-use watershed comprised of urban, suburban and agricultural land uses, resulted in nearly 500 *E. coli* isolates recovered over two years and different seasons across 100 different sites, but only one was an STEC [38]. Similar findings were observed in St. Clair and Detroit river areas where no STEC or EHEC were detected [39]. However, studies documenting the presence of *stx* virulence genes have reported higher prevalence of STEC. For example, six agricultural ponds in Central Florida were sampled for two growing seasons with each pond having either *stx1* or *stx2* detected at some timepoint (overall, *stx1*-32.6%, *stx2*-9.4%) [40]. As expected, in water sampled closer to agricultural areas dominated by cattle, a higher prevalence of STEC/EHEC has been noted. A study conducted in Australia looking at 6 diverse surface water sites ranging from locations in high-density urban areas to more rural areas subjected to mainly animal inputs found that of the 300 isolates recovered from 1L water grab samples, 16% were STEC, and EHEC recovery varied from 7% following a storm event to 11% during a dry period [41]. STEC recovery occurred at all sites but from the analysis completed, it is not clear if this was true of EHEC. A much higher prevalence has also been noted in highly polluted surface waters. For example, the Gomti River in India, which receives untreated domestic wastewater and had six distinct sites with varying amounts of recreation or bathing exposure, produced 90 isolates [42]. Of those 90 isolates, 40.1% carried either one or both *stx* genes and nearly 27% were EHECs. Also, a lesser appreciated area worth consideration is the impact of climate-driven increases in cyanobacteria and harmful algal blooms on STEC levels. This may be due to the synergistic cyanobacteria-bacterial interactions and the protection from UV effects and disinfectants algal mats may afford [43]. Nutrient increases following intense rainfall due to runoff and thermal warming and stratification of surface waters for longer durations boost the expansion and continuation of cyanobacteria and harmful algal blooms [43]. While the aquatic ecosystem is complex, there may be multiple, simultaneous effects on STEC survival due to associations with algal populations [44,45]. 

Water has played a documented role in human illness caused by the highly pathogenic *E. coli* O157:H7. Most notably, in the case of the significantly deadly Walkerton, Ontario outbreak in the year 2000 involving over 2000 confirmed cases [46] and more recently an outbreak involving toddlers with exposure to stream water next to a children’s playground [47], runoffs of animal feces were the suspected source. Few studies have specifically sought to isolate and identify *E. coli* O157:H7 from surface waters associated with agricultural areas. Presence of *E. coli* O157 was positively correlated with temperature and rainfall in all ten vegetable irrigation ponds sampled from the Suwannee River Watershed in Georgia over the course of the year-long study [48]. One comprehensive survey conducted in the Salinas and San Juan valleys of California, including the Salinas Valley watershed, sought to better understand the spread and persistence of O157:H7 strains in produce growing areas previously implicated in outbreak traceback investigations from 2002–2006 [49]. Over the 19 months that the Salinas Valley watershed was sampled, positive samples increased during periods of rainfall and in locations closer to cattle grazing with at least one strain of *E. coli* O157 being isolated from 6.5% of the samples collected. Likely watershed transport of up to 24 km was noted for some strains with all genetically indistinguishable isolates exhibiting a hydrological connection upon MLVA analysis. 

Ultimately, direct comparisons between the different studies of STEC prevalence and persistence are difficult due to differences in water sampling methodologies used, volumes of water collected, and methods of detection and identification (e.g., bacteriological agars used, gene detection by PCR versus cultural isolation). The isolation of STEC-positive colonies is very labor intensive and time consuming with many surveys reporting results of PCR screening of virulence genes (e.g., *stx*, *eae*) (e.g., [36,40]) which may overestimate the true prevalence of infectious organism since viability cannot be ascertained. Moreover, due to the availability of many easy tests to confirm O157:H7 compared to other STEC, surveys may choose to not capture the population of non-O157 EHEC. The lack of isolation also prevents whole genome sequencing to occur which makes it impossible to fully genetically characterize an isolate for comparisons between study isolates and any historical isolates. Additionally, investigations have noted *E. coli* O157 isolates from agricultural watersheds [48,50], without confirmation of H7. However, the true prevalence and risk to public health remains to be assessed since other H-types do not confer the same level of risk to human health.

### 2.2. Salmonella

*Salmonella* are Gram-negative, facultative, rod-shaped bacteria of the *Enterobacteriaceae* family. They are commonly found in the intestinal tracts of cold- and warm-blooded animals and considered enteric pathogens in humans. *Salmonella* can cause two major diseases: typhoid fever, characterized by high fever with little to no gastrointestinal illness, or non-typhoidal salmonellosis, characterized by gastrointestinal illness including severe diarrhea, abdominal cramps, and fever. Most cases of non-typhoidal salmonellosis result from ingestion of contaminated food or water. It is estimated that 93.8 million cases of salmonellosis occur yearly, in the U.S. and around the world, with approximately 155,000 deaths [51]. In the U.S., *Salmonella* infections result in close to $5.5 million in economic losses [52]. Recent Food Net data shows that the incidence of foodborne *Salmonella* infections is increasing despite increased efforts to control contamination events [2]. 

As a zoonotic pathogen, *Salmonella* infections are often associated with the contamination of animal products such as poultry and eggs, beef, pork, and fish. However, many illnesses are also associated with the consumption of fresh fruits and vegetables. From 2004 to 2012, there have been 71 and 40 outbreaks of salmonellosis linked to fresh produce in the United States and the European Union, respectively [53]. From 2010 to 2017, 56 multistate outbreaks of salmonellosis have been attributed to domestic and imported fresh produce including cucumbers, tomatoes, cantaloupe, papaya, and sprouts [24]. In 2020, *Salmonella* contamination of onions, wherein 1127 were sickened and 167 were hospitalized, was one of the largest foodborne outbreaks in the U.S. in recent times [54]. Finally, more alarming than the number of outbreaks associated with fresh produce, is the trend of higher hospitalizations and deaths associated with these outbreaks over outbreaks associated with meat, dairy or eggs [55].

There are multiple sources that can lead to fresh produce contamination along the farm to fork continuum. In the pre-harvest production environment, contaminated water used for irrigation, pesticide application and frost control, biological soil amendments, and dust/air are possible means of pathogen introduction [56,57]. Furthermore, contaminated water used for irrigation or other foliar applications, where water comes in contact with the edible portion of the plants, is of great concern. Studies involving flowering fruit and vegetable crops have demonstrated that exposure of the blossom to *Salmonella* often leads to externally and internally contaminated fruit [58,59,60]. For leafy vegetables, others have shown that *Salmonella* is able to enter the apoplast of the plant through the stomata [61,62]. Moreover, *Salmonella* has been shown to invade the root system of several plant varieties shortly after transplant, while the plant is still in transplant shock [58,59,60]. In all these instances, once *Salmonella* has internalized into the plant, post-harvest sanitation steps will not be able to reach or eliminate the pathogen. Therefore, understanding this pathogen’s biology and ecology in water is key to improving mitigation strategies to prevent pre-harvest contamination of produce due to contaminated water. 

Salmonellae are capable of extended survival outside of the intestinal tracts of its hosts. In a laboratory setting, *Salmonella* can survive for up to five years in sterile water at room temperature [63]. In natural aquatic environments, survival is harder to discern. In general, natural waters are harsh habitats due to low nutrient availability, temperature fluctuations, UV exposure, competition for available nutrients, and predation [64]. Sediments, biofilms, and association with green algal mats may offer protection in these environments [65,66,67,68,69]. Since the 1960s scientists have been studying the ability of *Salmonella* to survive in natural waters [70,71]. Hendricks and Morrison (1967) examined the presence of *Salmonella* in river water collected before and after a sewage treatment plant [71]. They also tested survival in the river itself by submerging dialysis tubing containing *Salmonella* cells, in order to expose the bacteria to the natural conditions of the river. Results showed small increases in cell number over a short period of time in the lab cultures and dialysis-sac study. Also, of note, extracts of river sediment had larger increases in cell numbers than just river water suggesting that along with protection, sediments may offer additional nutrients for survival [71]. In other studies, survival has been studied in microcosms of surface waters [72,73,74]. In these types of experiments the effects of excess nutrients, competition and predation were studied. The metabolic and genetic changes that the aquatic environment exerts on *Salmonella* are also yet to be discovered. Some studies have also suggested that *Salmonella* enters a dormant or viable but non-culturable state (VBNC) in aquatic environments as a mode of long-term survival [73,75]. Little is known about the mechanisms used to enter this state, how the cells remain viable in this state, or the exact mechanisms cells use to resuscitate from it [64]. The promise of next-generation sequencing technology may help to elucidate how these pathogens are surviving in an aquatic environment. For example, in a recent study using whole genome sequence comparisons between environmental and clinical strains, a difference was seen in the presence of the genes for D-galactonate degradation in environmental isolates [76]. The exact role that this pathway plays in environmental survival is yet unknown, but genomics, transcriptomics and metabolomics may be able to open new avenues to illuminate how this pathogen is able to survive in these harsh environments.

Multiple surveys have been conducted around the world looking at the prevalence, persistence and diversity of *Salmonella* in various watersheds. Most studies in the U.S. have focused on the East and West Coasts in areas where agricultural production is high. Along the East Coast, *Salmonella* prevalence ranges between 3% and 100% [4,5,8,40,77,78,79,80,81,82,83,84,85,86,87]. Most studies on the West Coast have been performed in CA, where prevalence ranges from 6% to 65% [6,88,89,90,91]. Studies outside the U.S. have found similar prevalence ranging from 23% to 78% [92,93,94,95,96,97,98,99]. In some of these studies, flowing water, such as rivers and creeks, are more likely to be positive for *Salmonella* than ponds or reservoirs [8,79,82,98]. Deavean et al. (2021) found that increased river discharge was the main variable associated with the presence of *Salmonella* in the Susquehanna watershed [77]. Likewise, Partyka et al. (2018) found that the flowing waters leading into and out of stratified lakes had a higher portion of *Salmonella* positives compared to the surface water layer of the lakes [90]. However, when comparing the surface, epilimnion layer, to the deeper, hypolimnion layer, more positive samples were found in the deepest portions of the lakes [90]. Several other studies have found more *Salmonella* positive samples in fresh water as compared to salt water [79,91,98]. 

Of course, the ultimate goal for most researchers, regulators and users of agricultural water is to identify an indicator that will easily mark a body of water as being contaminated with *Salmonella*. The indicators investigated have been biological, chemical or physical. Additionally, weather patterns have also been examined. Unfortunately, there has not been a lot of agreement in these studies. For biological indicators, generic *E. coli*, fecal coliforms, total coliforms, enterococci, and aerobic/heterotrophic bacterial plate counts have been compared to *Salmonella* presence/absence and concentrations. A few studies have identified a predictive relationship between the levels of generic *E. coli* and the probability of detecting *Salmonella* [81,82,87,100], while others have not seen this relationship [78,80,88]. The same dichotomy was seen with the other biological indicators [78,80,83,87,91,92,98,100]. Chemical indicators such as pH, conductivity, redox potential, dissolved oxygen and nitrogen/nitrate concentrations have also been studied for their ability to predict or correlate to the presence of *Salmonella*. The majority of the work has concluded that most of these indicators do not work well [80,81,83,91]. However, some have found correlations, such as Manan et al. (2020), who saw a positive correlation between nitrate-nitrogen levels and *Salmonella* presence but a negative correlation with dissolved oxygen and pH in waters along the mid-Atlantic region of the U.S. [8]. In contrast, Diaz-Torres (2020) found a positive correlation with conductivity, pH, and dissolved oxygen and *Salmonella* in water from Lake Zapotlan, Mexico [93]. Additionally, physical parameters of the water, such as temperature, salinity, and turbidity, have been explored. Again, with these parameters, there is no clear indication that they can predict or correlate to the detection of *Salmonella*. Some studies found water temperature to have a positive correlation with *Salmonella* presence [80,97], whereas others have found a negative correlation [91,93], or no correlation [77,83]. Similar results are seen for turbidity, where some have found positive correlations between turbidity and *Salmonella* [8,96], while others have found no correlation [80,83,91]. Like these biological, chemical and physical indicators, weather patterns, such as seasonality, rainfall, and air temperature have been investigated. Several research studies have found seasonality to play a role in the likelihood of detecting *Salmonella*, where more *Salmonella* positive water samples were found in warmer months typically of the summer and fall seasons [80,81,82,97,101]. Other studies did not necessarily see a seasonal effect in the ability to detect *Salmonella* but did see a seasonal effect on the serovar diversity [77,80,99]. For example, Thomas et al. (2013), saw more diversity in the summer and fall months compared to winter and spring [99]. Rainfall also has often been associated with an increased occurrence of *Salmonella* in water. Some studies have shown rainfall events 1–2 days before sample collection led to a greater propensity of isolating *Salmonella* from water [80,87]. Others have shown rainfall seven days before sample collection led to an elevated chance of finding *Salmonella* in water [82,87,91]. Gorski et al. (2011) found a correlation between the wet and dry seasons in CA, in that during the wet season there was a higher chance of recovering *Salmonella* from the water samples versus the dry season when recovery was less likely [89]. 

One major caveat that needs to be addressed when considering all of these studies is the different methods used to detect *Salmonella*, culturally or molecularly. While there are standard, validated methods for the culture, isolation and identification of *Salmonella* from foods [102], there are few standardized methods for complex environmental samples such as environmental and agricultural waters [103]. Most researchers have adapted foods methods for their individual studies but there is no consensus on the specifics for pre-enrichment, selective enrichment, or plating media. The most common pre-enrichment broths tend to be lactose broth [5,79,82], tryptic soy broth [6,88,89,91], buffered peptone water [80,84,85] and modified buffered peptone waters [4,77]. As seen in foods, where methods have recently been updated to move from pre-enrichment in lactose broth to universal pre-enrichment broth for spent sprout irrigation water [102], it is expected that the pre-enrichment broth will greatly affect the recovery of salmonellae from water. Likewise, the choice of selective enrichment and plating media may also greatly affect recovery. Analogously, incubation temperatures for each enrichment and plating step are also important for the recovery of *Salmonella*, where water may need to be treated as a high microbial background matrix and incubation temperatures adjusted accordingly [4]. Another issue to consider is that no matter which method is used there is some inherent enrichment bias that will favor the growth of 1 or 2 serovars, while masking any other serovars that may be present [104,105]. Newer molecular methods, such as CRISPR-SeroSeq, are shedding light on the full complement of serotypes present in a sample that may not always be isolated [105]. Finally, the use of molecular methods for the detection of *Salmonella* is appealing because of their fast detection time compared to the long, labor-intensive process of conventional microbiological culturing. However, as reviewed in Bell et al. (2016), water can be a complex and difficult matrix for molecular assays, such as PCR, due to the possible presence of inhibitors and/or high microbial background [106]. Caution should be used when designing, implementing and interpreting the results of these assays when analyzing water samples. 

### 2.3. Campylobacter and Emerging Arcobacter

The *Campylobacteraceae* family includes the *Campylobacter* and *Arcobacter* genera. Members of both genera are known to cause bacterial gastroenteritis worldwide and can be transmitted through contaminated food and water, contact with animal reservoirs, or person-to-person [107,108,109]. The gastrointestinal manifestations, including bloody diarrhea, are generally self-limiting. However, in rare cases, campylobacteriosis can result in inflammatory bowel disease (IBD), esophageal diseases, periodontal diseases, celiac disease, colorectal cancer, Guillain-Barré syndrome, Miller-Fisher syndrome, bacteremia and septicemia, cardiovascular complications, meningitis, and reactive arthritis [107]. The infectious dose of *Campylobacter* is thought to be low, as determined by human volunteer studies which showed that as few as 500 *C. jejuni* can cause disease [110]. 

Predominant sources of campylobacteriosis include undercooked poultry, raw milk and untreated drinking water [111]. Moreover, water can be the common source linking infection in humans, poultry, wild birds, and other domestic animals [112]. Waterborne outbreaks have been reported worldwide due to deficiencies in water treatment procedures, fecal contamination, discharge of wastewater into the water source, malfunctioning of the disinfection equipment or broken water pipes. It is worth noting that barring parasites, *Campylobacter* has been reported to be the predominant bacteria associated with waterborne outbreaks [113]. *Campylobacter* outbreaks associated with water date back to 1978 in the U.S. [114,115,116]. Overall, in the U.S., *Campylobacter* has been attributed to 12% of the waterborne disease outbreaks, and 78% of the overall acute gastrointestinal illnesses [117]. Data from waterborne outbreak investigations in Canada has shown *Campylobacter* to be one of the predominant causative agents [118]. As reviewed by Kaakoush et al. (2015), waterborne campylobacteriosis outbreaks have been reported in many developed countries in the last decade [107]. Among the developed nations, Nordic countries and Australia/New Zealand have reported higher waterborne *Campylobacter* outbreaks and cases, either due to water distribution failure or fecal contamination from wild birds [107,112,119,120,121,122,123,124,125,126]. Although there are reports of foodborne campylobacteriosis in Japan [107,127], there is only one report where the same strain of *C. jejuni* was found in patients with abdominal pain and water that was contaminated as a result of failure of chlorination [128]. The epidemiology of campylobacteriosis is difficult to elucidate in the developing world, mainly because of the ubiquitous nature of the pathogen in food sources and water, as well as confounding risk factors such as undernutrition/malnutrition, level of education, and lack of sanitation, thus making it difficult to make specific correlations [129,130,131]. However, studies have shown an association between drinking water and campylobacteriosis in children under the age of 5 in Northwest Ethiopia, the Northwest Province of South Africa, and in the rural coastal areas of Kenya [132,133,134]. 

*Arcobacter* spp. have been identified as emerging foodborne zoonotic pathogens worldwide and the International Commission on Microbiological Specifications for Foods has classified them as hazardous human pathogens. Although the role of *Arcobacter* species in causing human diseases is not fully established, three species including *A. butzleri*, *A. cryaerophilus* and *A. skirrowii* are predominantly associated with diarrhea, though many infections may be asymptomatic [108,135]. In a recent reclassification of the genus *Arcobacter* seven different genera were proposed of which *Aliarcobacter* gen. nov was described to include *Aliarcobacter cryaerophilus* comb. nov., *A. butzleri* comb. nov., *A. skirrowii* comb. nov., *A. cibarius* comb. nov., *A. thereius* comb. nov., *A. trophiarum* comb. nov., *A. lanthieri* comb. nov., and *A. faecis* comb. nov [136]. In this review we have indicated the species based on the original classification. There are several reports on the presence of *Arcobacter* in a wide range of waterbodies such as wastewater, seawater, lake water, river water, drinking water, groundwater and recreational water [137,138,139,140]. A massive waterborne outbreak on South Bass Island in Ohio was originally thought to be due to *Campylobacter*, but later *Arcobacter* was found in water from those wells to be associated with illness [141]. The first epidemiological connection between a water source with *Arcobacter* and diarrhea was made at a Girls Scout camp in Idaho in 1996 [142]. In an investigation comparing the environmental biotypes and serotypes of *Arcobacter* with those from clinical samples, researchers found that the strains isolated from water treatment plants were the same serotypes as in human isolates, suggesting that drinking water was the source of human arcobacteriosis [143]. However, in another waterborne outbreak in Finland, *Arcobacter* was isolated from water but not in fecal samples, possibly due to low levels of the bacteria in the clinical samples [123]. 

A comprehensive review by Pitkänen discusses waterborne *Campylobacter* outbreaks and the sources of water contamination. The review also suggests that *Campylobacter* diversity is based on the source of contamination, with sewage introducing *C. jejuni*, while bird feces introduce *C. jejuni*, *C. coli* and *C. lari* into water [122]. A variety of surface waters including rivers, lakes, water streams, and coastal waters can become contaminated with *Campylobacter* via animal and avian feces, agricultural run-off from farms, or sewage effluent, although ground water is less likely to be contaminated. For example, a significant positive association was observed between well-water prevalence and increased campylobacteriosis incidence in Maryland and Nebraska, with water contamination due to wastewater runoff from an adjacent concentrated animal feeding operation being the cause in the Nebraska outbreak [144,145]. Untreated or contaminated drinking water has been implicated in many outbreaks of campylobacteriosis. Furthermore, private water wells and rainwater tanks are added sources of waterborne campylobacteriosis [145,146,147,148,149,150]. As seen with *Campylobacter*, human pathogenic *Arcobacter* species have been isolated from sewage, wastewater (before and after treatment), waterbodies such as lake, river, spring and drinking water samples including groundwater [140,142,143,151,152]. More recently, *Arcobacter butzleri* was isolated from floodwaters after hurricane Florence in North Carolina, close to a region of swine and poultry production [153].

In an investigation carried out in South Africa, *A. butzleri* and several species of *Campylobacter* were isolated from surface water and sewage, while none were detected in ground or tap water [154]. Another study aimed at quantitatively detecting *Campylobacter* spp. over a 2-year period in Quebec, Canada showed that 43% of surface waters were positive for *Campylobacter* spp., whereas none of the groundwater wells, yet 10% of the private surface wells were positive for *C. jejuni* [155]. In another study, the prevalence of *Arcobacter* was assessed in water samples from different sources in Kathmandu Valley, Nepal where surface water was found to have the highest prevalence of *Arcobacter* compared to the other sources, without any seasonal differences [156]. Among the enteric pathogens, a higher prevalence of *A. butzleri* was also reported in water from shallow wells than boreholes in a sub-Saharan African region, especially during the wet season [157]. Overall, the prevalence of *Campylobacter* and *Arcobacter* is higher in surface than groundwater sources.

Heavy rainfall has been found to be associated with *Campylobacter* and/or *Arcobacter* contamination in various water sources, usually as the result of runoffs from nearby sources of animal fecal material. For instance, rainwater from a roof covered in bird feces flowed directly into drinking water reservoirs instead of into the rainwater drain, thus contaminating drinking water of a municipal distribution system in eastern Finland with *C. jejuni* [121]. Additionally, a higher association of campylobacteriosis was observed with increased rainfall in various regions of Ontario and Québec [118,121,158]. Similarly, extreme precipitation and a hurricane have resulted in contamination of water with *Arcobacter* on the South Bass island in Ohio and North Carolina, respectively [138,153]. The importance of water as a reservoir of *Arcobacter* is further suggested by their ability to survive for extended periods of time at various temperatures [142,159]. In addition to rainfall, some studies have reported a seasonal variation in the prevalence of *Campylobacter* species in environmental water, with increased detection in the Fall compared to Summer months, possibly reflecting a seasonal difference in *Campylobacter* shedding and discharge of fecal material into surface water and improved survival at lower temperatures (5-10°C) [149,155,160,161,162,163]. 

Studies have demonstrated that certain *Campylobacter* strains may survive in water for up to several months depending on the environmental conditions [164,165]. However, the ability to culture *Campylobacter* may be reduced when they become stressed under certain environmental conditions, including starvation and physical stress [122]. Despite being in an unculturable state, many of these *Campylobacter* cells may still be infectious [166]. Therefore, it is important to be able to detect, resuscitate and culture these stressed and infectious *Campylobacter*. Some countries have standard methods for the detection of *Campylobacter* spp. in water (US FDA BAM, ISO, Public Health England, Australia/New Zealand, and Norway), while other countries either adopt or adapt one or more of these protocols. Some similarities between these methods exist, including sample volumes of 100 mL to 4 L, a membrane filtration or centrifugation step to concentrate samples, and up to 48 h enrichment times, although there are more differences. The main differences are variations in the enrichment media (Bolton, Preston, Exeter), variations in the pre-enrichment and enrichment incubation temperatures and times, the application of a microaerobic growth environment, the addition of growth and/or antibiotic supplements, and the use of a secondary enrichment step. The similarities and differences of these standard protocols can have a significant impact on the ability to culture and detect *Campylobacter* from various water sources. For instance, earlier studies showed an improvement in *C. jejuni* isolation from river water with 37 °C enrichments, suggesting the possibility of false negatives with 42 °C enrichments [167]. However, more recently, Khan et al. reported greater recovery of *C. jejuni* and *C. lari* from surface water with a 42 °C enrichment while recovery of *C. coli* and other fastidious *Campylobacter* spp. was greater at 37 °C, suggesting that both enrichment temperatures may be required to maximize detection of all *Campylobacter* spp. [168]. The different combinations of antibiotics in enrichment broths can also affect the recovery of certain *Campylobacter* strains, depending on their antibiotic sensitivities. Previous studies have demonstrated that the elimination of antibiotics in the enrichment broth results in a reduction in *Campylobacter* growth due to the uninhibited growth of competing bacteria, making it essential to include antibiotics in the enrichment steps for *Campylobacter* isolation [169,170,171]. Others have explored alternative approaches to the use of antibiotics to improve the isolation of antibiotic-sensitive *Campylobacter*. For instance, use of filters on non-selective agar plates have been shown to prevent the passage of large and non-motile bacteria, while allowing the thin, motile *Campylobacter* cells through the filter to the agar [172,173,174]. However, this method works better when there are higher numbers of *Campylobacter* present as not all *Campylobacter* will pass through the filters, resulting in false negatives. 

Culture methods aimed at identifying thermotolerant *Campylobacter* spp. increase the possibility that some unculturable, infectious *Campylobacter* could go undetected in water samples, stressing the need for improved culture methods as well as the use of effective molecular methods. Some molecular methods, including PCR (standard, qRT-PCR, and multiplex PCR) and sequencing (16S rRNA and metagenomic analysis) have been developed or used independently or in concert with culture methods for the detection and characterization of *Campylobacter* in water samples [122,149,175,176]. A potentially useful tool for identifying viable, stressed *Campylobacter* cells is the viability quantitative PCR utilizing propidium monoazide (PMA-qPCR) to distinguish between dead and viable *Campylobacter* [177]. In addition, MALDI-TOF analysis has been used to isolate *Campylobacter* spp. from bird feces and river water in New Zealand [178].

Currently, there are no standard methods for the detection of *Arcobacter* in water. Further, while *Arcobacter* species are aerotolerant, conventional methods used for the isolation of microaerophilic *Campylobacter* can also enrich *Arcobacter* species [153]. Researchers from different countries have developed culture and molecular methods for the isolation and characterization of *Arcobacter* spp. in water. The different culture and molecular methods have been compared for specificity, selectivity, and reliability. These comparisons have shown that while these methods have been successful for isolating or identifying some of the *Arcobacter* spp. present in test samples, they are not 100% successful. For instance, direct plating was shown to be biased towards the recovery of *A. cryaerophilus* while enrichment prior to plating was biased towards recovery of *A. butzleri* [179]. Multiplex PCR assays were found to be more efficient than culture methods [180]. However, a comparison study of the performance of five different PCR assays aimed at identifying *Arcobacter* to the species level noted that none of the methods were completely reliable, with different identification rates ranging from 32.6–83.2% [181]. Improvements and alternate approaches in culture and molecular methods are still being developed. For instance, a chromogenic agar has been developed to isolate *A. butzleri*, *A. cryaerophilus*, and *A. skirrowii* [182]. Shrestha et al. developed a qPCR using primers designed against 16S rRNA to detect *Arcobacter* in various environmental water sources in Nepal [183], and Khan et al. developed a LAMP PCR to detect *Arcobacter* in agriculture and surface water samples [184]. The use of 16S rRNA RFLP is commonly utilized to characterize *Arcobacter* in water, despite its reported lack of reliability [179]. Recently, the use of 16s rRNA sequencing has become more common for characterization [140,179,185,186].

### 2.4. Listeria Monocytogenes

*Listeria* is a genus of Gram-positive bacteria which is ubiquitous in the natural environment [187]. Among the recognized species in this genus, *L. monocytogenes* is considered the only human pathogen and can be transmitted to humans via the food chain. The disease it causes, listeriosis, can be invasive or gastrointestinal [188]. Invasive listeriosis has a very high case-fatality rate, up to 20-30%, making *L. monocytogenes* a serious public health concern [189]. *L. monocytogenes* has been associated with foodborne outbreaks linked to contaminated dairy products, poultry, meat, seafood and produce [190,191]. Notably, quite a few recognized listeriosis outbreaks in recent years were linked to contaminated produce such as stone fruits, caramel apples, leafy green salad, cantaloupes, frozen vegetables and sprouts [8,192,193,194,195].

*L. monocytogenes* and non-pathogenic *Listeria* spp. have been frequently isolated from irrigation and natural waters. This could be due to their ubiquitous presence in natural environments, such as soils, native vegetation, animal feed and feces [6,196,197]. Qing et al. (2018) sampled pond and river waters in Maryland and Pennsylvania during the produce growing season (from March to July) [192]. *Listeria* spp. were found in 27% of pond water samples and 100% of river water samples, while *L. monocytogenes* was found in 22% of the pond water samples and 98.5% of the river water samples. Using a metagenomics sequencing approach, Gu et al. (2020) surveyed four vegetable farms along the Eastern Shore of Virginia and found *L. monocytogenes* in 27% of the pond water samples and 4% of the well water samples [198]. Likewise, Sharma et al. (2020) identified *L. monocytogenes* in 31% of samples from six non-traditional irrigation water sites [8]. Other surveys conducted in New York state, in various water sources, found *Listeria* spp. in 16 to 58% of the samples analyzed [195,196,199], and *L. monocytogenes* in 28 to 51% of the samples [195,196,200]. In two separate regions of CA, *L. monocytogenes* was found in 43 and 62% of the waters investigated [6,201]. Waters sources in Canada were found to have a lower prevalence of *L. monocytogenes*, with numbers ranging from 10 to 22% [193,194,202,203]. Across the globe, others have found *L. monocytogenes* in various watersheds ranging from 4 to 53% [197,204,205,206,207,208]. A few studies have quantitatively assessed the concentration of *L. monocytogenes* in water samples, which were typically low. A longitudinal study conducted in PA reported concentrations of this pathogen between ≤2.1 MPN/L to 561 CFU/L [193]. Waters from vegetable farms in the Eastern Shore of Virginia contained 6.44 MPN/L or less of *L. monocytogenes* [198]. The levels of *L. monocytogenes* in six non-traditional irrigation water sites in the Mid-Atlantic U.S. were less than 1 MPN/L except for one site where up to 5 MPN/L was observed. Interestingly this site had a lower water temperature than other sites [8].

*Listeria* contamination in water appears to be strongly affected by temperature. Collectively, studies have shown greater occurrence and concentrations of *L. monocytogenes* in the cooler winter season than those found in the warmer summer season [6,8,193,194,200,202]. The psychrotrophic nature of *Listeria* may offer it a competitive advantage over other mesophilic microbes in these natural environments [187]. Another very interesting observation is the tendency to find *L. monocytogenes* serotype 1/2a more often in cooler temperatures than serotype 4b [203,208]. It should be noted that other variables could confound these analyses of temporal/seasonal effects on *L. monocytogenes* prevalence. For example, in certain geographic regions winter also has significant rainfalls and summer caused changes in water flows [193]. Also, seasonal variations in agricultural activities may affect the seasonal distribution of *L. monocytogenes* [203]. Thus, comprehensive and in-depth multivariate analyses of multiple factors should be performed in order to fully understand the effects of environmental and climatological factors on the occurrence of *L. monocytogenes* in different water sources.

Other factors, such as pH, turbidity, proximity to animals, rainfall and salinity of sea water, that affect the contamination of *Listeria* in waters, have been discussed. Qing et al. (2018) found that *L. monocytogenes* levels were higher in river waters compared to pond waters in the Mid-Atlantic region of the U.S. [192]. Another study on the mid-Atlantic water sites had similar observations, and the authors suggested that lower *L. monocytogenes* levels in the pond and reclaimed waters can be possibly due to these water bodies undergoing some treatments to remove contaminants [8]. In the water samples around the produce production environments in New York State, *Listeria* and *L. monocytogenes* were higher in water samples collected from sources not used for irrigation (e.g., roadside ditch, runoff ditch) than in samples from sources used for irrigation (e.g., well or municipal waters, pond); and when combining water samples from all sources *Listeria* spp. and *L. monocytogenes* were most frequently isolated from surface water samples (e.g., ponds, rivers, and creeks) compared with well or municipal water samples [194,195]. Water samples from produce production environments were more likely to be contaminated with *Listeria* than water from a nonagricultural environment [195]. In the study of five fruit and vegetable farms in New York State, being close to pastures, cattle and dairy farms, and impervious surfaces were shown to affect the occurrence of *L. monocytogenes* in a water sample [196]. All *L. monocytogenes*-positive water samples were from surface waters (e.g., creek or pond water), and not from engineered water sources (e.g., municipal or well water) [196]. In contrast, Sauders et al. (2012) found higher incidence of *Listeria* spp. in surface water samples from urban environments than those in natural environments of New York State [199]. This difference could be due to variations in agricultural practices, drainage, landscape attributes, urban development, water management and other geographic factors [197,198,200]. The study on the watersheds in Nova Scotia, Canada found that incidence of *L. monocytogenes* was not related to storm events [197]. Interestingly, the study on the South Nation River in Ontario, Canada found that higher rainfalls were associated with reduced occurrence of *L. monocytogenes* [203]. In contrast, increased occurrence of *Listeria* in water samples in Austria was associated with rainfall and flooding events; flooding events were also associated with high diversity of *L. monocytogenes* genotypes [197]. One study of surface waters in the mid-Atlantic region of the U.S. observed the largest concentrations of this pathogen in the water following the precipitation events; the author speculated that an increase in *L. monocytogenes* could be attributed to soil runoff into water following precipitation events as well as an increase in flow rate in the creek that leads to the perturbation of the sediment and the release of *Listeria* into the water [209]. The study on California Central Coast watersheds also reported that the high incidences of *L. monocytogenes* corresponded to high precipitation [6]. In the study of the South Nation River watershed of Ontario, there was a significant association between the occurrence of *L. monocytogenes* and proximity to an upstream dairy farm and degree of cropped land [203]. The study on water samples in western Switzerland also showed the association between *L. monocytogenes* serotype 4b and *L. ivanovii* presence and cattle presence; however, the same study did not find such correlation between the presence of *L. monocytogenes* serotype 1/2a and cattle [208]. The study on the British Columbia water samples suggested that *L. monocytogenes* presence correlated with upstream livestock [193]. The same study found that most watersheds that had alkaline pH and pH closer to neutral was associated with higher incidence of *L. monocytogenes* [193]. In contrast, the study on 12 water sites in Austria showed that incidence of *Listeria* spp. was highest at pH 7.94 (range 7.2 to 8.87) [24,197]. Gu et al. (2020) reported positive correlation between water turbidity and *L. monocytogenes* occurrence and thus hypothesized that soil and possibly other external sources might have introduced *L. monocytogenes* into water [198]. *Listeria* was found in estuarine water, although the prevalence of *Listeria* was reduced when the salinity was increased, and recovery rate dropped to zero when salinity was 15 g/L [206]. In the study of the irrigation canal of two rivers in Mpumalanga, South Africa, chemical oxygen demand positively correlated with the presence of *L. monocytogenes* [204].

Individual species of *Listeria* spp. and specific genotypes of *L. monocytogenes* in waters were identified in a few studies. Such analyses also shed light on the possible source of contamination. All *Listeria* spp. including *L. innocua* [195,196,197,199,206], *L. ivanovii* [194,197,206,208], *L. seeligeri* [195,196,197,199,206], *L. welshimeri* [199,206], and *L. grayi* [192] have been isolated in waters. Overall, there is no clear indication that one species dominates in waters, although significant associations between certain species and certain water sites were observed [192,193,194,196,197]. *L. monocytogenes* isolates from irrigation pounds in Maryland had relatively high genetic diversity including genetic lineage I (22.4%), II (37.5%), and III (36.9%) [210]; this study identified twelve novel clones of *L. monocytogenes*, and none of these water strains matched strains from recent U.S. outbreaks. The waters in two watersheds of Nova Scotia, Canada had high incidence of serogroup IIa (i.e., serotypes 1/2a, 3a), followed by IVb (i.e., serotypes 4b, 4d, 4e), IIb (i.e., serotypes 1/2b, 3b) and IIc (i.e., serotypes 1/2c, 3c) [194]. One survey of the surface waters in the mid-Atlantic U.S. found *L. monocytogenes* genetic lineage I (21.2%), II (48.2%), and III (31.6%) [211]. The strains did not match strains from recent U.S. outbreaks; however, some strains belonged to multilocus sequence typing (MLST) clonal complexes (CCs) (CC1, CC4 and CC6) that have been strongly associated with clinical cases and are considered hypervirulent [212], suggesting water as a reservoir for *L. monocytogenes* strains that could cause human illnesses or outbreaks. Over 85% of isolates in watershed sites in the California Central Coast agricultural region belonged to serotype 4b with other isolates belonging to serotypes 1/2a, 1/2b and 3a [6]. In one of the studies on South Nation River watershed of Ontario, Canada, 50% of the *L. monocytogenes* isolates belonged to serogroup IIa and 32% of the *L. monocytogenes* isolates belonged to serogroup IVb, and overall genetic lineage I (i.e., including serogroups IVb and IIb) and genetic lineage II (i.e., including serogroups IIa and IIc) isolates were equally abundant when the prevalence for the whole year was analyzed [203]. In the mountainous surface and groundwaters in western Switzerland, *L. monocytogenes* serotypes 1/2a and 4b were predominant, while serotype 1/2b was less frequent [208]. In the study on the 12 geological and ecological sites in Austria, 27 *L. monocytogenes* isolates belonged to 16 MLST sequence types (STs), indicating high clonal diversity, previously identified hypervirulent clones, ST1/CC1, ST2/CC2, ST4/CC4 and ST6/CC6, were isolated from these water samples [197]. Similarly, many *L. monocytogenes* isolates recovered from the South Nation River watershed in Ontario, Canada matched human clinical isolates in the Canada PulseNet database [203]. The *L. monocytogenes* population from the irrigation waters in British Columbia were serotypes 4b and 1/2a; the authors speculated that the wild animals could be a source of contamination since serotype 4b isolates had been isolated from wild animals [193]. The large percentage of lineage III isolates from water samples reported in one of the surveys of the mid-Atlantic U.S. water samples also suggested a possible link to animals [210,211] since genetic lineage III isolates of *L. monocytogenes* were often found in animals [213].

A limited number of studies attempted to determine the correlation between background flora or indicator organisms and *Listeria* or *L. monocytogenes*. In a study using a metagenomics approach, *Rhizobacter* was positively correlated with the occurrence of *L. monocytogenes*, which may be due to run off from plant rhizosphere soil into irrigation waters [198]. Macarisin et al. (2018) evaluated the correlation between levels of *E. coli*, *Enterococcus* and *L. monocytogenes* in surface waters used for irrigation and reported a very weak correlation between *L. monocytogenes* counts and *E. coli* and enterococci concentrations in river waters [214]. In the watersheds of Nova Scotia, Canada, elevated *E. coli* levels were associated with a higher likelihood of detecting *Listeria* spp. but were not related to the incidence of *L. monocytogenes*; this was very interesting since *E. coli* is widely used as an indicator for fecal contamination in water [194]. Similarly, the study on the South Nation River in Canada found no or negative associations between *L. monocytogenes* and water quality indicator bacteria such as *E. coli* and coliforms among different seasons [202]. The study on Eastern Shore of Virginia irrigation ponds also showed that fecal indicators did not significantly correlate with *L. monocytogenes* incidence [198]. Indeed, considering that surface waters are commonly inhabited by *L. monocytogenes*, which originates from a variety of natural reservoirs such as soil, decaying vegetation and fecal contamination, microbiological quality standards for agricultural waters should probably rely on factors in addition to bacterial fecal indicators. The role of human or animal input on *L. monocytogenes* concentrations in surface waters is also yet to be fully understood, and thus, a better understanding of the major sources of this pathogen in agricultural natural waters is needed. Therefore, new markers or indicator organisms need to be identified and validated for the assessment of microbiological quality of agricultural waters.

While *Listeria* spp, and *L. monocytogenes* were frequently isolated from waters in different studies, different qualitative and quantitative methodologies were used, and to date no comprehensive comparison has been performed to evaluate the performance or efficacy of recovery for these different methods. Protocol differences include varying enrichment broths, incubation temperature and times, the use of secondary enrichments and immunomagnetic separation to try to enhance the detection of *L. monocytogenes* [6,8,193,194,195,196,197,198,199,202,203,206,207]. Enrichment schemes have been shown to significantly affect the recovery rates of heavily stressed *Listeria* [215]. Specifically, enrichment schemes with up to 48 h duration vastly outperformed those with up to 24 h duration [37,215]. The FDA Bacteriological Analytical Manual calls for streaking of selective agar plates not only at 48 h but also at 24 h [216]. Streaking at 48 h ensures that *L. monocytogenes* has fully recovered and been allowed to grow to detectable levels. Streaking at 24 h may enhance the recovery when competing background microflora may outgrow *L. monocytogenes* in the enrichment broths and on the selective agars. For two-step enrichment schemes, the volume of culture transfer from primary enrichment to secondary enrichment is critical due to *L. monocytogenes*’ very slow growth during the first 24 h of enrichment. Once sufficient enrichment incubation duration was achieved, the selection of enrichment broths appeared to be not critical as long as an extensively validated enrichment broth was chosen [215,217]. 

Another confounding variable, when studying *Listeria* biodiversity in waters, is that oftentimes *L. monocytogenes* and multiple other *Listeria* species coexist in waters. To date *Listeria* selective enrichment broths will enrich all major *Listeria* species, so the dominant species in a water sample could outgrow the other species including *L. monocytogenes*. This is especially possible when one species is 1-2 logs higher than the other species [218,219,220]. Stea et al. (2015) reported a very high percentage of water samples presumptive positive for *L. monocytogenes* by PCR but negative by a culture-based method, and the authors hypothesized that *L. innocua* and other *Listeria* spp. may have outcompeted *L. monocytogenes* during the selective enrichment [194]. This issue becomes important when studying the correlation between *Listeria* spp. and *L. monocytogenes* in a specific water site to evaluate whether *Listeria* spp. is a good indicator for *L. monocytogenes*. In addition, specific enrichment schemes might introduce bias towards certain serotypes or sequence types [221]. For instance, Gorski et al. (2014) compared enrichment schemes with and without a selective enrichment in Fraser broth using water samples and found that serotype 1/2a strains were more likely to be isolated with the use of Fraser broth [222]. One way to circumvent these culture-based issues is to test multiple presumptive colonies to enhance the isolation of multiple genotypes possibly present in one sample. Also, if the purpose is to specifically detect *L. monocytogenes*, chromogenic agars that utilize cleavage of substrates by virulence factors may be preferred [223]. For quantitative analysis, the relatively low concentrations of *Listeria* spp. and *L. monocytogenes* in surface waters makes accurate enumeration challenging. Chen et. al. (2017) demonstrated that the employment of non-traditional MPN schemes in combination with 48 h enrichment in Buffered *Listeria* Enrichment Broth was highly efficient for the detection of very low levels of *L. monocytogenes* in naturally contaminated food samples [224]. Similar non-traditional MPN schemes, with a lower limit of detection, can be employed for the enumeration of *L. monocytogenes* in surface waters.

## 3. Antimicrobial Resistance (AMR) in Environmental Waters

Antimicrobial compounds have been widely used to control and prevent bacterial infection in humans, animal husbandry, crop production and aquaculture. Although antibiotics and bacterial AMR genes (ARGs) are considered natural components of the microbial communities in different ecosystems, human impacts have drastically changed their ecology. The increasing rise of AMR has become one of the top global threats to public health [225,226]. Human, livestock, soil, manure, and wastewater treatment plants are major reservoirs of antimicrobial agents, their metabolites, antimicrobial-resistant bacteria (ARB) and their genes (ARG) [227,228,229,230,231,232,233]. Environmental water serves as an important conduit for the introduction and dissemination of AMR among humans, animals and plants/crops, as drinking water and irrigation water often originates from surface water, which is also the discharge point for wastewater. Recreational activities and agricultural runoff can also contaminate surface water, contributing to the transmission of AMR. 

Antibiotics and ARG in environmental waters and their adverse public health effects have been well substantiated within the scientific literature. A few studies have also characterized the prevalence of antimicrobial-resistant pathogenic and non-pathogenic bacteria including *Salmonella*, *E. coli*, and *Enterococcus* in surface water in different regions of the world [82,84,234,235,236,237,238,239,240,241,242,243,244,245,246,247,248,249,250,251,252,253,254,255]. However, most studies are limited in scope such as sampling sites, frequency, or targets for analysis. A direct comparison between studies is often difficult since different isolation or enrichment methods were used for these environmental bacteria, and different antimicrobial drugs were used to assess the antimicrobial susceptibility. Nevertheless, these ARB can be prevalent and persistent in environmental water and resistant to a wide range of antimicrobials [84,234,239,240,241,242,243,244]. Increased prevalence of these environmental ARB was found in surface water receiving discharge sewage from animal farms, wastewater treatment plant, hospitals, and community, thus increasing the risk to public health [256,257]. A global surveillance of AMR using metagenomic analyses was performed recently to analyze bacterial resistomes in urban sewage collected from 79 sites in 60 countries [258]. ARG abundance was reported to correlate strongly with socio-economic, health and environmental factors. In the study, ARGs encoding resistance toward macrolides, tetracyclines, aminoglycosides, beta-lactams, and sulfonamides were the most abundant, with a high relative proportion of macrolide resistance genes in most samples from Europe and North America and a large proportion of genes providing resistance to sulfonamides and phenicols found in Asian and African samples [258]. The metagenomics approach may provide a standardized way for continuous global surveillance of ARB and ARGs. The National Antimicrobial Resistance Monitoring System (NARMS), established in 1996, has been monitoring the ARB with public health importance, including *Salmonella*, *E. coli*, and *Entercoccus*, isolated from humans, retail meats and food animals in the United States. To establish a One Health AMR monitoring system, NARMS added environmental monitoring of surface water into the current model for combating ARB through collaboration with the U.S. Environmental Protection Agency (EPA) [259]. This particular effort will standardize the methods, including microbiological, targeted gene quantification, and metagenomics methods used in the field, and associated metadata, and provide a national-scale, quantitative assessment of AMR within surface water [260].

## 4. The Role of Genomics in Detection, Traceability, and Characterization of Enteric Bacterial Pathogens Associated with Water

### 4.1. Whole Genome Sequencing (WGS)

Whole genome sequencing (WGS) represents the newest and perhaps most pivotal technology now at the disposal of field and food safety scientists focused on the microbiological aspects of surface and agricultural waters. WGS is the term denoted for the sequencing of the entire genomic DNA of a bacterial pathogen. It is the ultimate subtyping tool and uses massively parallel robotic sequencing technology to provide all of the genetic information that distinguishes one bacterial strain from another [261,262]. The applications of WGS are manifold and include outbreak detection and characterization, source-tracking, determining the root cause of a contamination event, profiling virulence and pathogenicity attributes in a strain, antimicrobial resistance monitoring, and quality assurance for microbiology testing, just to name a few [263,264,265]. Since being applied retrospectively for the first time ever in a foodborne outbreak event in 2009 for source-tracking *Salmonella* in a spiced salami outbreak [262] and then piloted as a network in 2012, again in *Salmonella*, during a sushi outbreak [266], WGS has been a mainstay for molecular epidemiological surveillance and traceback for food safety investigators and scientists focused on exploring root causes of produce-borne contamination events across the U.S. and abroad.

FDA’s GenomeTrakr WGS network and database was also established around this time. The GenomeTrakr is an open-source whole-genome sequencing network of state, federal, academic, and commercial partners focused on application of pathogen genome sequencing and comparison to delimit the scope and temporal window of an outbreak event as well as to better pinpoint and understand the sources, reservoirs, and vehicles for pathogen distribution during a contamination event [261,267,268,269]. The GenomeTrakr network represents a first-of-its-kind distributed genomic food shield for characterizing and tracing foodborne pathogens back to their sources, enabling longitudinal study microbiologists to immediately query their sequence to a WGS database of nearly a million genomes of food, environmental, and clinical pathogen sequences for potential linkages and leads. The GenomeTrakr network is supporting outbreak investigations with unprecedented accuracy in microbial surveillance and traceability and allows for immediate and effective monitoring of good agricultural practices and preventive controls for produce production and processing environments. 

Applications of WGS in the study of enteric pathogens associated with agriculturally destined surface waters are manifold, often used to fill three specific knowledge gaps associated with pathogens relevant to the farm environment. The primary application of using WGS for pathogen surveillance in surface and agricultural water has been to look for close matches between these pathogen genomes and clinical isolate genomes in the GenomeTrakr database, although any WGS linkage can support an investigation and direct additional inquiry [267,270,271]. Comparisons of this nature permit inquiry into whether any isolates present in surface waters have caused downstream human illness presumably through the consumption of a readily-consumed-raw produce commodity such as lettuce. Second, in addition to finding similar clinical “matches” in the database, WGS data provides a strong evolutionary signal and can inform environmental strain traceability, enabling the identification of a recent common ancestor or potential source reservoir such as feral animals, birds, veterinary agriculture sources or even human encroachments [266,272]. Akin to the one-health paradigm, knowledge discovered using WGS evidence directly links food, human, animal and environmental isolates and helps our understanding of the sources and mechanisms of pathogen contamination and helps guide preventive controls to minimize or prevent pathogens from contaminating food on the farm [4,106]. Finally, WGS data can provide insight into whether a particular growing region retains an endemic, single, highly fit, pathogenic, environmental clone and whether that clone is persistent in the environment or is being constantly reintroduced from an external source. In further support of this latter application, careful inspection of WGS data can often times reveal specific adaptations or genome acquisitions that infer additional fitness for specific strains endemic to a region [273]. 

**Figure 1:**
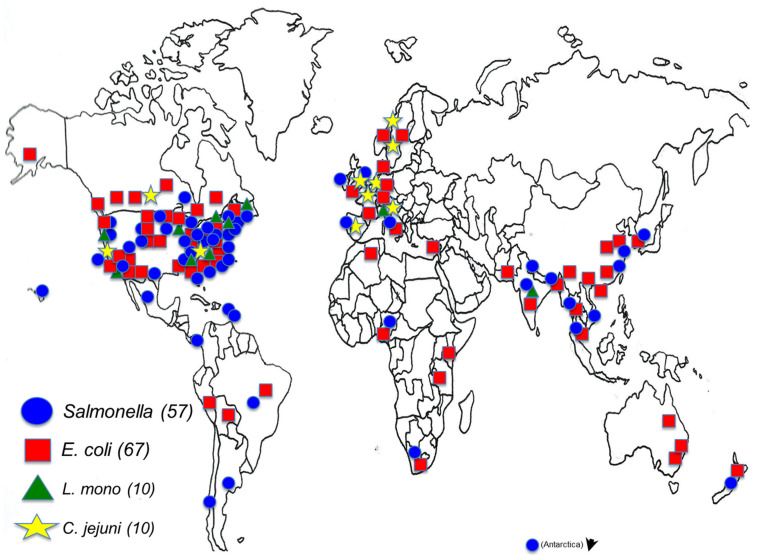
GenomeTrakr global distributions of several foodborne bacterial species isolated from various water sources. Geographic locales, highlighting water-associated strains by country or state, are plotted in global relief in order to provide an overall distribution of several foodborne species genomes currently available in the GenomeTrakr Network Database at the NCBI Pathogen Detection portal. Each separate shape represents one or more strains for that specific species from that specific locale (country, province, or state). Shapes denote foodborne bacterial species as follows: circles, *Salmonella enterica*; squares, *E. coli/Shigella*; triangles, *L. monocytogenes*; and stars, *C. jejuni*. Parentheticals at the end of each species name denote the total number of pinpoints marked for the species indicated. Entries were extracted from the “Location” metadata field linked to each GenomeTrakr/NCBI submission (https://www.ncbi.nlm.nih.gov/pathogens/ accessed on 8 March 2021).

The GenomeTrakr/NCBI Pathogen WGS database currently contains more than three-quarters of a million entries with about 600,000 pathogen genomes originating from the four major foodborne bacterial species: *S. enterica*, *E. coli* and *Shigella spp*., *L. monocytogenes*, and *C. jejuni* (https://www.ncbi.nlm.nih.gov/pathogens/ accessed on 08 March 2021). Astonishingly, despite the epidemiological and ecological importance of surface and other waters in foodborne contamination, water-associated GenomeTrakr submissions for these four pathogens comprise less than 1.5% (n = 8497) of the total database with water-associated genomes of *Salmonella*, *E. coli*, *L. monocytogenes*, and *C. jejuni* representing 1.7%, 1.1%, 2.4%, and 0.4%, respectively, for each of these pathogen’s sub-databases. Despite the overall dearth of water related genome submissions, metadata inspection of these entries revealed a wide variety of disparate isolation sources including sediments, ponds, streams, rivers, creeks, lakes, bays, oceans, environmental and agricultural reservoirs, canals and rinse water collectors to name only a few. *Salmonella* submissions alone retained more than 34 specific water-related niches. Commiserate with observed diversity among isolation source, genome submissions for all salmonellae represented 90 different named serovars and subspecies along with numerous additional antigenic formulas for which a name has yet to be assigned. Finally, a geographical overlay of submission sources reveals several interesting findings (Figure 1). First, most submissions associated with some form of water appear to originate from the U.S., Canada, or Western Europe. However, total water submission counts from China, India, and SE Asia were noteworthy. Surprisingly, a paucity of representative water-related genomes was observed from the Southern hemisphere, including many agriculture-rich areas of South America and Africa. This last observation underscores the importance of establishing global water surveillance initiatives with and among scientists from these important regions. The JIFSAN/FDA Water and Food Safety Consortium currently supports a collaborative fusion of genomics, surface waters, and enteric pathogen sampling among food safety and field scientists from several Latin And South American nations, providing one example of such partnerships to strengthen pathogen surveillance and expansion in global surface waters [274].

### 4.2. Metagenomics

One form of WGS called metagenomics is also bringing us closer to culture independent diagnostic characterization of foodborne pathogens by identifying and sequencing all associated genomes, including pathogens, in a single environmental sample such as surface water. Analysis of metagenomic sequence data allows for detection of multiple serotypes or serovars of a pathogenic species within a single sample, which can guide pathogen recovery attempts. Metagenomic analysis has recently been targeted for several foodborne pathogens in select ecological niches (i.e., rivers and canals and other potential agricultural water niches around the farm) and in environmental matrices such as STECs in agricultural waters. Moreover, longitudinal studies, such as those described here, are accelerating the development of the MetagenomeTrakr Network of Laboratories (a metagenomic-specific database within the GenomeTrakr Network) focused on providing microbial fingerprints including virulence, contamination, spoilage, and biogeographical microbial signatures of food and food ecologies such as agricultural waters, soils, phytobiomes, compost, and dust. These can be used for pathogen source- tracking and baseline pathogen profiling to respond to specific farm-related public health needs [275]. 

### 4.3. Long-Read Sequencing 

Of particular interest right now, is the emerging area of “long-read” whole genome sequencing, which can rapidly and accurately produce closed genomes of bacterial species in situ. Long read metagenomic sequencing using Oxford Nanopore Minion handheld sequencing technology, as one example, is both rapid and portable for potential use in farm-based environmental studies involving surface waters and sediments as well as other complex environmental matrices. A recent study [276] documented these new methods to detect and assemble STEC and EHEC directly from field irrigation water. They also determined the limits of detection and identification of STECs by Nanopore long-read sequencing from pre-enriched field irrigation water artificially contaminated with *E. coli* O157:H7. *E. coli* O157 could be detected at low levels (103 CFU/mL), and an *E. coli* O157:H7 full genome was also obtainable from this sample allowing for in silico virulence detection. Maguire et al. (2021) further characterized the background bacterial species in the pre-enrichment, including antimicrobial resistance genes, which could have important implications to farm and water safety [276]. They found that some of those species carried important ARGs and were also potential human pathogens (e.g., Klebsiella pneumoniae). These novel long-read WGS methods may provide enhanced environmental surveillance applications for actionable and timely decisions on the presence of STECs and other pathogens in agricultural waters and throughout the farm environment. Indeed, a combination of this data flow and methods could be deployed with environmental microbiology experts in the field to generate assembled genomes for pathogens found commonly in environmental sample types of interest (e.g., soil, cow manure, compost, and especially water).

## 5. Global Initiatives

### 5.1. Latin American Water Study

WGS has been broadly used to provide detailed characterization of foodborne pathogens. Applications in food safety using WGS approaches include outbreak detection and characterization, source tracking, determining the root cause of a contamination event, profiling of virulence and pathogenicity attributes, AMR monitoring, quality assurance for microbiology testing, as well as many others. FDA established the GenomeTrakr network in 2013 with the goal to build a global one health WGS database where human pathogens are rapidly characterized and linked to closely related food and environmental isolates for the rapid investigation of illnesses/outbreaks. A pilot program to engage international partners for isolating and sequencing *Salmonella* from surface water was initiated with university collaborators from Chile and Mexico in 2018. The sample collection began in March 2019. The project has received additional funding and been expanded to include two universities in Brazil in 2019–2020. Despite the Covid-19 pandemic, the project has made impressive progress. More than 1700 water samples were collected, and 813 *Salmonella* isolates were sequenced. 

The two universities in Chile, Universidad Chile and Universidad Andres Bello, have partnered and obtained 1140 samples in 38 visits to four rivers. The isolation rates of *Salmonella* ranged from 8.3% to 45%, with an average of 28%. A 300% increase in isolation rate was detected in southern rivers in warm months compared to cold months. A total of 642 isolates of *S. enterica* from water samples in Chile have been sequenced. Serotype and AMR of each isolate were predicted based on the WGS data. There were 123 antimicrobial-resistant *S. enterica* isolates. The National Autonomous University of Mexico processed 324 surface water samples from 86 different sampling points (rivers, dams, ponds, lakes, and irrigation canals) from May of 2019 to October 2019. Out of these samples, 254 were *Salmonella* positive. An additional 130 samples were collected in 2020 across 74 different sampling points. The overall prevalence of *Salmonella* was approximately 80%. Among 171 *Salmonella* isolates sequenced from Mexico, 137 were predicted to be antimicrobial-resistant. The two Brazilian universities (Federal University of Paraiba and Federal University of Rio de Janeiro) joined the project in August 2020. A total of 60 samples from small rivers and irrigator canals near farms were collected in the State of Rio de Janeiro in 2020. Approximately 60% of the samples were positive for *Salmonella*. In Paraíba State, Northeastern Brazil, 42 water samples were collected in January 2021 and 7 (16%) samples were positive for *Salmonella*.

This study provided a comprehensive analysis of *Salmonella* in Latin American surface waters associated with produce production. The data will aid in the expansion of the global WGS database, further validate environmental sampling and analysis methods, assess the distribution and subtypes of *Salmonella* in these waters, and provide insight into the proficiencies and barriers faced by other nations in these efforts. 

### 5.2. Global Water and Food Safety Summit

International experts gathered in College Park, MD November 19–21, 2019 for the inaugural Global Water and Food Safety Summit to discuss the important intersection of water and food safety. Sponsored by the U.S. Food and Drug Administration’s Center for Food Safety and Applied Nutrition and The University of Maryland Joint Institute for Food Safety and Applied Nutrition, this first-of-its-kind event provided a platform to better understand how water impacts the safety of our food supply and the scientific approaches used to shed light on these connections [274]. 

The goal of this meeting was to assemble a variety of international experts in the field to address the impact, importance, and challenges of microbiological sampling of water for food safety and public health. The magnitude of this summit incorporated detailed general sessions with multiple break-out meetings, which drew over 180 people from 17 countries to attend this 3-day conference. The experts and professionals represented 23 universities, 23 corporations and originations, and 7 federal and local governmental agencies and the United Nation’s Food and Agricultural Organization (FAO). The summit provided an excellent forum for the participants to engage in the formation of new and important collaborations, promote global data sharing as part of a global open source WGS database, understand the root causes and potential environmental sources of produce contamination as well as contribute to our greater understanding of the risks of pathogen contamination of fresh and fresh cut produce farm systems across the U.S. and around the world. This meeting was the first of hopefully many such discussions.

## 6. Advances in Methods and Preventive Measures

### 6.1. Water Collection Methods

As mentioned with each of the pathogens above, the laboratory methods utilized play a critical role in the efforts to isolate and identify each organism in water samples. Water sample collection is also crucial for the effective detection of these pathogens. These enteric microorganisms are not expected to be present at high numbers in natural and agricultural waters [80,87,198,209,277]. As such the volume of water tested will affect the culture results. For example, in a study associated with three waterborne *Campylobacter* outbreaks in Finland, water samples were analyzed using volumes ranging from 4 to 20 L and found that the chance of detecting *Campylobacter* increased with increasing sample volumes [120]. In another example, Sharma et al. (2020) reported that sampling 10 L of water was 43.5 and 4.8 times more likely to find *Salmonella* and *L. monocytogenes* (respectively) than testing 1 L [8]. 

Water sampling methods range from simple grab samples to complex filtrations (Table 1). The easiest method is the simple grab sample, which typically allows for 100 mL to 1 L of water to be collected. Larger volumes may be collected depending on the size of the container available. One major disadvantage of this method is in the transport of the samples back to the laboratory, where large coolers with ice and tight sealing containers, to avoid leakage, are needed. Once back in the lab these samples may be directly (e.g., no concentration) assayed or they may undergo further processing, such as centrifugation or membrane filtration, before enrichment [4,40,97]. Because large (10 L) to very large (100 L) volumes of water may need to be collected, on-site, field deployable filtration methods have been explored. The simplest of these methods include the Moore swab (reviewed in [278]), where microbes are captured in cheesecloth over extended periods of time. Fernandez et al. demonstrated that the use of Moore swabs placed in a river for 24 h resulted in more *Campylobacter* positive samples compared to membrane filtration of 4 L water samples from the same river [279]. And Benjamin et al. (2013) reported greater recovery of *Salmonella* from CA waterways using a Moore swab deployed for 3 to 5 days versus grab samples [88]. A major advantage of the Moore swab is due to its extended deployment in the body of water to allow for greater capture of the microbes that may only be intermittently present. However, the method requires the sampling team to place the swab and then return day(s) later to recover it. An extension of the Moore swab is the modified Moore swab (mMS), where a set volume of water is pumped through a tightly rolled ‘swab’ of cheesecloth [10,280,281,282]. To date the mMS has only been thoroughly evaluated for filtering 10 L of water [282], which can easily be achieved in about 30 min on site by the sampling team. For much larger volumes, the use of hollow fiber ultrafilters, with a molecular weight cutoff of approximately 30 KDa, in a tangential or dead-end configuration have been assessed [283,284,285]. These ultrafilters have a pore size 45X smaller than standard 0.45 μm filters and a much larger surface area, 2.5 m^2^ or 1440X larger than a 47 mm filter [285]. Because of these attributes, much larger volumes of water can be filtered while ensuring the complete capture of all microbes present in the filtered sample. It should be noted, though, along with capturing the microbiota, all particles larger than the pore size are also retained, including soil/sediment particles, dissolved organics, other larger organic or inorganic particles, etc., which may interfere with downstream testing, especially molecular assays such as PCR, for the desired pathogens [285]. Additionally, the turbidity level of the water will affect the volume of water that may be successfully filtered. In general, in the tangential configuration the filters are less likely to foul and clog due to the scrubbing effect of the water passing along the filter fibers [283]. However, this set-up is not easily field deployable and requires the transport of the retentate fluid back to the lab, again necessitating the use of large coolers and tight-sealing containers [283,285]. To alleviate these issues, using the dead-end configuration allows for a single pass filtration, where only the filter needs to be transported back to the lab. Furthermore, studies have shown that significant fouling of the filter resulting in a reduction of the flow rate occurred after 72 L of high turbidity (92NTU) water had been filtered [285] (Mull 2012). The use of dead-end ultrafiltration demonstrated that low levels (10 CFU/60 L) of *Campylobacter* could be recovered from spiked and naturally contaminated water samples [277], and as few as 50 O157:H7 cells from 40 L of spiked water [286]. 

### 6.2. Development of Preventive Measures

Agricultural water has been identified as a major risk factor in the contamination of produce. Surface water represents one of the riskiest water sources [287,288]. Therefore, mitigation strategies need to be taken to reduce the risk of produce contamination with pathogens when using surface water for agricultural application. Besides actively monitoring water quality, treating water physically and/or chemically during storage and while in the delivery system is also a feasible strategy to reduce the risk of produce contamination. However, there are currently no registered antimicrobial treatment products that are authorized to control microorganisms of public health significance for use on agricultural fields, or for treatment of irrigation water systems or ponds. In April 2020, EPA approved and published, with FDA, a disinfectant efficacy protocol for chemical companies to use to test their products against foodborne pathogens in preharvest agricultural water [289]. Disinfectants have been used in agricultural settings in various applications including sanitation, equipment cleaning and disinfection, plant pathogen control, and algae control. This work now provides the growers a legal pathway forward to be able to control human foodborne pathogens in their surface and irrigation waters on the farm. However, multiple factors need to be taken into consideration in field application of preharvest agricultural water treatment. The principal factors that influence disinfection efficiency are disinfectant concentration, contact time, temperature and pH [290]. Increased resistance to disinfection may also result from attachment or association of microorganisms to particles that cause turbidity [291,292], and algae [293]. Additionally, enteric pathogens may form biofilms on environmental substrates which also contributes to increased resistance to disinfection [294,295]. Therefore, combining pretreatment of water, such as removal of algal cells and removal of turbidity by sand filtration and other filtration methods, together with a proper disinfection scheme may provide efficient removal of pathogenic contaminants from agricultural water.

## 7. Conclusions

Into the future, a changing climate and related extreme weather events (e.g., intensified precipitation events, prolonged droughts) will no doubt have some impact on pathogen prevalence and persistence in global surface waters. Warmer water temperature could increase adaptive aquatic populations while amplified rainfall may mean more runoff of pathogens, like those discussed here, from adjacent lands entering water used for crop irrigation. Moreover, those waters may exceed capacity and excess contaminated water may flood onto nearby land used for food production. In sum, environmental research focused on surface and agricultural waters as potential root causes of contamination of fresh and fresh-cut produce will always be an essential element to responding to produce-borne outbreaks. A strong focus on water is and will remain a key component to any environmental scientific effort. With newly developed and adapted collection and sampling technologies, including those described here, studies on water will continue to provide extensive insight into the ecological drivers of pathogen contamination on the farm, and their resultant findings will be imperative to better understand the many ways in which fresh produce can become contaminated with enteric pathogens in the field. Furthermore, global engagement is necessary. The scientific collaborations, such as those mentioned here, will foster future partnerships. International guidance is needed to increase the awareness on how the combination of classical microbiology and WGS has helped to investigate foodborne disease in which water plays a role.

## Figures and Tables

**Table 1:** Comparisons of Water Collection Methods.

Method	Ease of Use ^a^	Field Deployable	Easy to Transport	Volume Sampled	Cost ^b^
Grab	easy	Yes	no	100 mL to 1 L ^c^	$
Moore swab	easy	Yes	yes	NA	$
modified Moore Swab	intermediate	Yes	yes	10 L	$$
Tangential Filtration	complicated	not readily	no	10 L-100 L	$$$
Dead End Ultrafiltration	intermediate	Yes	yes	up to 100 L	$$$

^a^ Categories: easy: does not require any complex equipment or training to deploy; intermediate: requires some equipment and/or simple training; complicated: requires equipment and training. ^b^ $: $1–$10, $$: >$10, $$$: >$50 per sample collected. ^c^ Volume of a grab sample may be larger and is limited by the size of the container used.

## Data Availability

Not applicable.
